# Birth preparedness and complication readiness among women of child bearing age group in Goba woreda, Oromia region, Ethiopia

**DOI:** 10.1186/1471-2393-14-282

**Published:** 2014-08-18

**Authors:** Desalegn Markos, Daniel Bogale

**Affiliations:** Department of Nursing, College of Medicine and Health Sciences, Madawalabu University, Bale, Goba Ethiopia; Department of Public Health, College of Medicine and Health Sciences, Madawalabu University, Bale, Goba Ethiopia

**Keywords:** Birth preparedness, Complication readiness, Goba woreda, Ethiopia

## Abstract

**Background:**

Birth preparedness and complication readiness is the process of planning for normal birth and anticipating the actions needed in case of an emergency. It is also a strategy to promote the timely use of skilled maternal care, especially during childbirth, based on the theory that preparing for childbirth reduces delays in obtaining this care. Therefore, the aim of this study was to assess birth preparedness and complication readiness among women of child bearing age group in Goba woreda, Oromia region, Ethiopia.

**Methods:**

A community based cross sectional study was conducted in Goba woreda, Oromia region, Ethiopia. Multistage sampling was employed. Descriptive, binary and multiple logistic regression analyses were conducted. Statistically significant tests were declared at a level of significance of P value < 0.05.

**Results:**

Only 29.9% of the respondents were prepared for birth and its complications. And, only 82 (14.6%) study participants were knowledgeable about birth preparedness and complication readiness.Variables having statistically significant association with birth preparedness and complication readiness of women were attending up to primary education (AOR = 3.24, 95% CI = 1.75, 6.02), attending up to secondary and higher level of education (AOR = 2.88, 95% CI = 1.34, 6.15), the presence of antenatal care follow up (AOR = 8.07, 95% CI = 2.41,27.00), knowledge about key danger signs during pregnancy (AOR = 1.74, 95% CI = 1.06,2.88), and knowledge about key danger signs during the postpartum period (AOR = 2.08, 95% CI = 1.20,3.60).

**Conclusions:**

Only a small number of respondents were prepared for birth and its complications. Furthermore, the vast majority of women were not knowledgeable about birth preparedness and complication readiness. Residence, educational status, ANC follow up, knowledge of key danger signs during pregnancy and the postpartum period were independent predictors of birth preparedness and complication readiness.

## Background

Globally, maternal mortality remains a public health challenge [1]. World Health Organization (WHO) estimated that 529,000 women die annually from maternal causes. Ninety nine percent of these deaths occur in less developed countries. The situation is most dire for women in Sub-Saharan Africa, where one of every 16 women dies of pregnancy related causes during her lifetime, compared with only 1 in 2,800 women in developed regions [2].

The global maternal mortality ratio (MMR) decreased from 422 (358-505) in 1980 to 320 (272-388) in 1990, and was 251 (221-289) per 100 000 live births in 2008. The yearly rate of decline of the global MMR since 1990 was 1.3% (1.0-1.5). More than 50% of all maternal deaths were in only six countries in 2008 (India, Nigeria, Pakistan, Afghanistan, Ethiopia, and the Democratic Republic of the Congo) [3]. In Ethiopia, according to 2011 Ethiopia Demographic and Health Survey (EDHS) report, MMR remains high which is 676 deaths per 100,000 live births. However, only 10% of births in Ethiopia occur in health facility while 90% women deliver at home. Reasons for not delivering at health facility for more than six women in ten (61%) stated that a health facility delivery was not necessary, and three in every ten (30%) stated that it was not customary [4].

Birth preparedness and complication readiness (BP/CR) is a comprehensive package aimed at promoting timely access to skilled maternal and neonatal services. It promotes active preparation and decision making for delivery by pregnant women and their families. This stems from the fact that every pregnant woman faces risk of sudden and unpredictable life threatening complications that could end in death or injury to herself or to her infant. BP/CR is a relatively common strategy employed by numerous groups implementing safe motherhood program in the world [5, 6].

In many societies in the world, cultural beliefs and lack of awareness inhibit preparation in advance for delivery and expected baby. Since no action is taken prior to the delivery, the family tries to act only when labor begins. The majority of pregnant women and their families do not know how to recognize the danger signs of complications. When complications occur, the unprepared family will waste a great deal of time in recognizing the problem, getting organized, getting money, finding transport and reaching the appropriate referral facility [7].

It is difficult to predict which pregnancy, delivery or post delivery period will experience complications; hence birth preparedness and complication readiness plan is recommended with the notion of pregnancy is risk [6]. BP/CR strategy encourage women to be informed of danger signs of obstetric complications and emergencies, choose a preferred birth place and attendant at birth, make advance arrangement with the attendant at birth, arrange for transport to skilled care site in case of emergence, saving or arranging alternative funds for costs of skilled and emergency care, and finding a companion to be with the woman at birth or to accompany her to emergency care source. Other measures include identifying a compatible blood donor in case of hemorrhage, obtaining permission from the head of household to seek skilled care in the event that a birth emergency occurs in his absence and arrange a source of household support to provide temporary family care during her absence [6, 8].

With different effort from government as well as nongovernmental organization working on maternal issues, pregnant women were not found to be well prepared for birth and its complication. For example, only 47.8% women who have already given birth in Indore city in India [9] and 35% of pregnant women in Uganda were prepared for birth and its complication [10]. Additionally, according to the research done in some part of Ethiopia, only 22% of pregnant women in Adigrat town [7] and 17% of pregnant women in Aleta Wondo of southern region [11] were prepared for birth and its complication. Even though there are some studies which were conducted on this similar issue in Ethiopia, they mainly encompass the urban area. Additionally, there are some differences in socio demographic and cultural conditions of these different regions of the country. Therefore, the aim of this study was to assess birth preparedness and complication readiness among women of child bearing age group in Goba woreda, Oromia region, Ethiopia.

## Methods

### Study area and period

The study was conducted in Goba woreda, Bale zone from April 1-28, 2013. Bale zone is administratively divided in to woreda and smaller kebele. Goba woreda is one of the woreda in Bale zone, Oromia region of Ethiopia and located 444 km from Addis Ababa. Currently, the woreda has 24 rural and 2 urban kebeles. Based on figures obtained from 2007 census, this woreda has an estimated total population of 73,653; of whom 37,427 were females and 32,916 (44.7%) of the population were urban dwellers [12]. The estimated total number of women of reproductive age and pregnant women in the woreda were 16,277 and 2725 respectively. The woreda have one health post in each kebele, 4 health centers, one Hospital and Blood Bank. One ambulance from hospital as well as Red Cross society serves the community in providing transportation service from home to the health facility ( [13], Goba woreda health office: Annual health report, unpublished]).

### Study design

A community based cross sectional study was conducted. Women who gave birth in the last 12 months regardless of their birth outcome were included in the study and those women who were severely ill, mentally and physically not capable of being interviewed were excluded from the study.

### Sample size and sampling procedure

#### Sample size

The study employed single population proportion sample size determination formula. Twenty two percent proportion (p) of birth preparedness and complication readiness [7] with 95% CI, and 5% marginal error (where n is desired sample size, Z is value of standard normal variable at 95% confidence interval and, p is proportion of Birth preparedness and complication readiness and d is marginal error which is 5%) was considered to calculate the sample size. Since multistage sampling was employed, the calculated sample was multiplied by 2 for design effects to control the effect of sampling that could happen due to using sampling method other than simple random sampling, and 10% contingency for non respondents were also added. After all, the final sample size became 580**.**

### Sampling procedure

Multistage sampling was employed to select study subjects. First, all kebeles in the woreda were stratified in to urban and rural. Goba Woreda constitutes 24 rural kebeles and one town administrative (consisting of two urban kebeles) that makes up a total of 26 kebeles. To determine representative sample of kebeles for the woreda and got adequate sample, 1/3^rd^ of the kebeles were selected. Based on the above calculation, 9 kebeles were chosen using simple random sampling from the total 26 kebeles. According to the strata i.e. urban and rural residence, a kebele from the 2 urban and 8 kebeles from the 24 rural kebeles were selected using simple random sampling technique. Then, the total sample size (n =580) was allocated proportionally to the size of the selected kebeles. Finally, systematic sampling was employed to select the study subjects in each kebele until the desired numbers of sample was obtained. To select the first house hold in each kebele, first a land mark which is common in almost all kebeles that is health post was identified. A pen was spin and the direction pointed by the tip of the pen was followed. To select the first house hold, one of the house which was included under the initial sampling interval of each kebele was selected by simple random sampling; lottery method. Then, the next house hold was selected through systematic sampling technique that is every K^th^ interval household which was calculated for each kebele because the numbers of households vary from one kebele to another kebele. In a case when the study participants were not be able to be interviewed for some reason (e.g. absenteeism), attempt was made for three times to interview the respondent and after all, they were considered as non respondents. On the other hand, if the household did not include women who meet the inclusion criteria, the next household was substituted. Moreover, if the household contained more than one candidate, one of them was taken randomly by employing lottery method.

### Operational and term definitions

Birth preparedness and complication ready: A woman was considered as prepared for birth and its complication if she identified four and more components from birth preparedness complication readiness item [7].

Skilled provider: are persons with midwifery skills (Physicians, Nurses, Midwives, and Health Officers) who can manage normal deliveries and diagnose, manage or refer obstetric complications.

Knowledge on key danger signs of pregnancy: a woman was considered as knowledgeable if she spontaneously mentioned at least three key danger signs of pregnancy otherwise not knowledgeable.

Knowledge on key danger signs of labor: a woman was considered as knowledgeable if she spontaneously mentioned at least three key danger signs of labor otherwise not knowledgeable.

Knowledge on key danger signs of post partum: A woman was considered knowledgeable if she spontaneously mentioned at least two out of the three key danger signs of post partum period otherwise not knowledgeable.

Knowledge of birth preparedness and complication readiness: A woman was considered knowledgeable if she can spontaneously mentioned at least 4 item of knowledge of birth preparedness and complication readiness question otherwise not knowledgeable.

### Data collection tool and procedure

Validated structured questionnaire adapted from the survey tools developed by JHPIEGO Maternal Neonatal Health program was used. Information on socio demographic characteristic was collected. Additionally, knowledge of key danger signs during pregnancy, delivery and postpartum period and its actual problem which require referral were asked. Furthermore, the study subjects were asked their BP/CR practice waiting their spontaneous answer to check whether they practiced those operationally defined BP/CR component. These were identifying place of delivery, plan of skilled assistant during delivery, saving money for obstetric emergency, plan of mode of transport to place of delivery during emergency, plan of blood donor during obstetric emergency, detecting early signs of emergency and identifying institution with 24 hour emergency obstetric care services. Eight diploma Nurses who are fluent in speaking Amharic and Afan Oromo were involved in the data collection. They collected the data through face to face interview. In addition to the principal investigators, four Bachelor of Science degree (BSc) holder health professionals were recruited and supervised the data collection process.

### Data quality control

The Qualities of data were assured by using validated questionnaire, translation, retranslation and pretesting of the questionnaire. The questionnaire was translated from English language to Amharic and Afan Oromo by a translator and back to English language by second other translators who were health professionals to compare its consistency. The pretest was done on 5% of the total sample size in Robe town. Content and face validity were checked by reproductive health expert. Additionally, after the pretest, to check the internal consistency of the tool, cronbach alpha value was calculated and its value for birth preparedness and complication readiness item was 0.86. Data collectors and supervisors were trained for two days on the study instrument and data collection procedure. The principal investigator and the supervisors strictly followed the overall data collection activities.

### Data processing and analysis

The data were checked for its completeness and consistencies. Then, it was cleaned, coded and entered in to computer using statistical package for social sciences (SPSS) windows version 16.0. Descriptive statistics were computed to determine the prevalence of birth preparedness and complication readiness. Moreover, binary logistic regression analysis was performed to identify those factors associated with birth preparedness and complication readiness. Then, to control the effect of possible confounders, multiple logistics regression was computed with a confidence interval of 95%. P value < 0.05 on a binary logistic regression was considered to select candidate variable for multiple logistic regression analysis as well as to declare statistically significant variable.

### Ethical consideration

The proposal was approved by Ethical Review Committee of College of Medicine and Health Sciences of Madawalabu University. Furthermore, letter of permission was obtained from Bale zone health department and Goba woreda health offices. Consent was obtained from the study subjects after explaining the study objectives and procedures and their right to refuse not to participate in the study any time they want was also assured.

## Results

Out of the total 580 women planned for the study, 8 (1.4%) study subjects refused to respond for the question,10(1.7%) individuals were not found in three times visit and 562 were successfully interviewed yielding the response rate of 97%.

### Socio demographic characteristics

The mean age of the respondents were 26.6 (SD ± 5.9) year. Majority of respondents, 33.8%, were between the age group of 21 and 25 years and a few were in the age group of less than 20 years. Muslim and Orthodox Tewahido were the dominant religions each accounting 49.1%. Most participants, 266 (47.3%), reported that they were educated up to primary school followed by those who did not attend formal education; 161 (28.6). Four hundred ten (73%) respondents were Oromo in their ethnic group, followed by Amhara; 139 (24.7%). The vast majority, (85.2%), of respondents were housewives. Regarding the marital status of study subjects, 531 (94.5%) of them were married. The mean family size and monthly income of the participants were 5.0 (SD ± 2.1) and 1267.9 (SD ± 1298.7) Ethiopian Birr respectively. About 224 (39.9%) study subjects did not have any financial income (Table 1).

**Table 1:** **Socio-demographic characteristics of the respondents, Goba woreda, Oromia region, Ethiopia, April, 2013**

Variable	Frequency	Percent
**Residence**		
**Urban**	116	20.6
**Rural**	446	79.4
**Age**		
**≤** **20**	94	16.7
**21-25**	190	33.8
**26-30**	164	29.2
**>30**	114	20.3
**Mean (** **±** **SD)**	26.6 (±5.9)	
**Religion**		
**Muslim**	276	49.1
**Orthodox**	276	49.1
**Protestant**	7	1.2
**Other** ^*^	3	0.5
**Marital status**		
**Married/in Union**	531	94.5
**Single**	14	2.1
**Widowed**	8	1.4
**Divorced**	6	1.1
**Separated**	3	0.5
**Ethnicity**		
**Oromo**	410	73
**Amhara**	139	24.7
**Tigre**	2	0.4
**Gamo**	2	0.4
**Other** ^**^	9	1.6
**Educational level**		
**None**	161	28.6
**Read and write**	16	2.8
**Primary**	266	47.3
**Secondary and above**	119	21.2
**Occupation**		
**Housewife**	479	85.2
**Gov’t. employee**	21	3.7
**Private employee**	14	2.5
**Merchant**	42	7.7
**Other** ^***^	6	1.1
**Monthly income of the women** ^**+**^		
**None**	224	39.9
**(None-100]**	56	10
**101-300**	135	24
**≥** **301**	147	26.2
**Total family income** ^**+**^		
**≤** **100**	12	2.1
**101-300**	60	10.7
**≥** **301**	487	87.1
**Mean (** **±** **SD)**	1267.9 ( ± 1298.7)	
**Family size**		
**≤** **4**	281	50
**5-6**	162	28.8
**≥** **7**	119	21.1
**Mean (** **±** **SD)**	5.0 (±2.1)	

*indicate Catholic and Wakefeta, **indicate Welayita, Gurage, ***indicate daily laborer.

^+^Currency is measured in Ethiopian Birr.

### Birth preparedness and complication readiness

**Figure 1:**
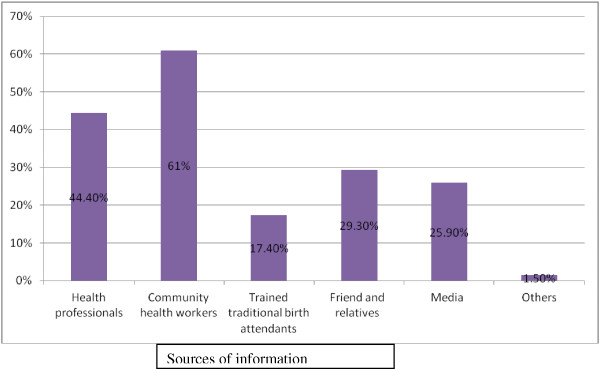
**Sources of information about birth preparedness and complication readiness as reported by study participants in Goba woreda, Oromia region, Ethiopia, April, 2013.** N.B. This cannot be sum up to 100% because multiple responses were possible.

Out of the total respondents, 301 (53.6%) study participants reported that they have never heard the term birth preparedness and complication readiness. Their sources of information for the majority of respondents, 61.0%, were community health workers (Figure 1).

Among those respondent who knew about birth preparedness and complication readiness, 415 (87.6%) spontaneously identified and mentioned preparing essential items for clean delivery and post partum period followed by saving money; 330 (69.6%) (Table 2). In summary, only 82 (14.6%) study subjects were knowledgeable about birth preparedness and complication readiness.

**Table 2:** **Knowledge of respondents about preparation for birth and its complication, Goba woreda, Ethiopia, April, 2013**

S. N**o**	Variable	Frequency	Percent (N = 474)
1	Identify place of delivery	214	45.1
2	Save money	330	69.6
3	Prepare essential items for clean delivery & post partum period	415	87.6
4	Identify skilled provider	34	7.2
5	Being aware of the signs of an emergency & the need to act immediately	16	3.4
6	Designating decision maker on her	4	0.8
7	Arranging a way to communicate with a source of help	33	2.7
8	Arranging emergency funds	67	5.5
9	Identify a mode of transportation	25	5.3
10	Arranging blood donors	15	3.2
11	Identifying the nearest institution that has24 hours functioning EmOC services	58	12.2

N.B. This cannot be sum up to 100% because multiple responses were possible.

Generally, only 168 (29.9%) study subjects were prepared for birth and its complication in their last pregnancy where as the remaining 394 (70.1%) study subject did not (Table 3).

**Table 3:** **Practices of respondents on preparation for birth/complication, Goba woreda, Oromia region, Ethiopia, April, 2013**

S. No	Variable	Frequency	Percent
**1**	Identify place of delivery	432	76.9
**2**	Plan skilled assistant during delivery	297	52.8
**3**	Saved money for obstetric emergency	388	69
**4**	Plan a mode of transport to place of delivery during emergency	353	62.8
**5**	Plan blood donor during obstetric emergency	51	9.1
**6**	Detect early signs of an Emergence	177	31.5
**7**	Identified institution with 24 hr EmOC services	267	47.5

N.B. This cannot be sum up to 100% because multiple responses were possible.

### Factors associated with birth preparedness and complication readiness

On binary logistic regression, place of residence, occupation, educational level, family size, ANC follow up, knowledge of danger sign during pregnancy, labour and postnatal period, as well as gravida and parity were found to have statistically significant association with birth preparedness and complication readiness.

Multiple logistic regression analysis was also computed to control the possible confounder, explores the association between selected independent variables, and birth preparedness and complication readiness. The odds of birth preparedness and complication readiness were two times greater among urban resident when compared to rural resident (AOR = 2.01, 95% CI = 1.20, 3.36). Additionally, educational level of mother was also found as a predictor of birth preparedness and complication readiness. The odds of birth preparedness and complication readiness of woman who attended to the primary, and secondary and higher level of education were three and nearly three times higher than that those who did not attend formal education (AOR = 3.24, 95% CI = 1.75, 6.02) and (AOR = 2.88, 95% CI = 1.34, 6.15) respectively. Furthermore, the odds of birth preparedness and complication readiness were eight times greater among women who have ANC follow up when compared with women who did not have ANC follow up (AOR = 8.07, 95% CI = 2.41,27.00). Besides, the odds of birth preparedness & complication readiness among knowledgeable women about key danger signs during pregnancy were nearly two times greater than not knowledgeable women (AOR = 1.74, 95% CI = 1.06,2.88). Similarly, the odds of birth preparedness and complication readiness among knowledgeable respondents about key danger signs during postpartum period were two times greater than those who lack knowledge about it (AOR = 2.08, 95% CI = 1.20,3.60) (Table 4).

**Table 4:** **Association of selected socio-demographic and obstetric factors of respondents with preparation for birth and its complication, Goba woreda, Oromia region, Ethiopia, April, 2013**

Variable	Practice of birth preparedness & its complication		COR (95% CI)	AOR (95% CI)
	Not prepared (%)	Prepared (%)		
**Residence**				
**Urban**	57 (49.1)	59 (50.9)	3.2 (2.09,4.88)	2.01(1.20,3.36)
**Rural**	337 (75.6)	109 (24.4)	1	1
**Marital status**				
**In marital union**	370 (69.7)	161 (30.3)	1.49(0.63, 3.53)	
**Not in marital union**	24 (77.4)	7 (22.6)	1	
**Occupation**				
**Housewife**	349 (72.9)	130 (27)	1	1
**Gov’t. employee**	9 (42.9)	12 (57.1)	3.57 (1.47, 8.69)	1.39(0.48,4.04)
**Private employee**	9 (64.3)	5 (35.7)	1.49 (0.49, 4.53)	1.15(0.33,4.01)
**Merchant**	24 (57)	18 (42.9)	2.01 (1.05, 3.83)	1.45(0.68,3.07)
**Other** ^***^	3 (50)	3 (50)	2.68 (0.53, 13.4)	1.69(0.22,13.11)
**Age**				
**≤** **20**	70 (74.5)	24 (25.5)	1	
**21-25**	125 (65.8)	65 (34.2)	1.51 (0.87, 2.63)	
**26-30**	119 (72.6)	45 (27.4)	1.10 (0.62, 1.96)	
**>30**	80 (70.2)	34 (29.8)	1.24 (0.67, 2.28)	
**Educational level**				
**None**	144 (89.4)	17 (10.6)	1	1
**Read and write**	12 (75)	4 (25)	2.82 (0.81, 9.73)	1.96(0.49,7.79)
**Primary**	173 (65)	93 (35)	4.55(2.59, 7.99)	3.24(1.75,6.02)
**Secondary and above**	65 (54.6)	54 (45.4)	7.03 (3.79, 13.06)	2.88(1.34,6.15)
**Total family income**			1	
**≤** **100**	11 (91.7)	1 (8.3)	3.34(0.39, 28.24)	
**101-300**	46 (76.7)	14 (23.3)	5.03(0.64, 39.37)	
**≥** **301**	334 (68.6)	153 (31.4)		
**Family size**				
**≤** **4**	177 (63.9)	100 (36.1)	2.35 (1.40,3.95)	1.19(0.46,3.04)
**5-6**	117 (72.7)	44 (27.3)	1.57 (0.88,2.78)	1.22(0.61,2.46)
**≥** **7**	96 (80.7)	23 (19.3)	1	1
**ANC follow up**				
**Yes**	313 (65.5)	165 (34.5)	14.23(4.42, 45.75)	8.07(2.41,27.00)
**No**	81 (96.4)	3 (3.6)	1	1
**Knowledge status of danger signs during pregnancy**				
**Not knowledgeable**	92(51.4)	87 (48.6)	1	1
**Knowledgeable**	302(78.9)	81 (21.1)	3.52 (2.40, 5.16)	1.74(1.06,2.88)
**Knowledge status of danger signs during labour**				
**Not knowledgeable**	321(78.3)	89 (21.7)	1	1
**Knowledgeable**	73 (48)	79 (52)	3.90 (2.62, 5.79)	1.66(0.97,2.84)
**Knowledge status of danger signs during postnatal period**	336 (76.7)			
**Not Knowledgeable**	58 (46.8)	102 (23.3)	1	1
**Knowledgeable**		66 (53.2)	3.74(2.47, 5.68)	2.08(1.20,3.60)
**Gravid**				
**1**	103 (64)	58 (36)	1.86(1.17,2.96)	0.32(0.05,2.02)
**2-3**	142 (68.6)	65 (31.4)	1.51 (0.97, 2.36)	0.39(0.09,1.61)
**≥** **4**	149 (76.8)	45 (23.2)	1	1
**Parity**				
**First**	109 (64.5)	60 (35.5)	1.90 (1.19, 3.0)	2.64(0.38,18.17)
**Second**	76 (61.8)	47 (38.2)	2.14 (1.29,3.54)	3.91(0.81,18.90)
**Third**	67 (77)	20 (23)	1.03 (0.56,1.90)	1.39(0.46.5.62)
**Fourth and above**	142 (77.6)	41 (22.4)	1	1

## Discussion

This study showed that, only a small proportion of the respondents were prepared for birth and its complication in their last pregnancy. The finding is not in agreement with the result of the study done in rural Uganda, Mbarara district [10]. On the other hand, the result is almost consistent with another study done among pregnant women in Aleta Wondo Woreda, Sidama zone, Southern Ethiopia [11]. Besides, it is slightly in agreement with the finding of study conducted in Adigrat town, North Ethiopia [7]. The difference with the finding of the study done in rural Uganda, Mbarara district could be unlike the current study, in the former study there was a mixture of study subject where pregnant women in addition to women who gave birth in the last 12 months were considered. So, pregnant women might tell their future plan rather than what they have actually performed. On the top of this, majority of the respondent in rural Uganda, Mbarara district attended ANC for four and more visit where they could have more opportunity to get information concerning birth preparedness and complication readiness than the current study subject where only a few women attended four and more ANC visit.

The most commonly mentioned practice in the study were identifying place of delivery, saving money which may be explained by the fact that both women and their partners may knew that money is required to facilitate referral in case of complications, planning skilled assistant and identifying institution with 24 hour emergency obstetric care. It is nearly comparable with community based study in rural Uganda, Mbarara district where majority of the respondents identified a skilled providers, saved money, identified means of transport, and bought delivery kits/birth materials during their most recent pregnancy [10].

In contrary to the practice of BP/CR among women, only a small number, 82 (14.6%), of study participants were knowledgeable about birth preparedness and complication readiness. The implication of this finding could be women could practice some of those BP/CR components without having the knowledge of its rationale. Therefore, their continuous practice in the future is under question because of their knowledge gap.

Regarding some of the factors affecting birth preparedness and complication readiness, the study found educational status of the women, and ANC follow up has significant statistical association with birth preparedness and complication readiness. This finding is also supported by another community based study done in Adigrat town, North Ethiopia [7]. The implication of this finding could be when a women become educated, they might have better access for information from different sources like from reading different materials and for the case of ANC follow up, if the women have ANC follow up, they could accept advise and health information from health professionals. So that helps them be prepared for birth and its complication.

As a strength, this study tried to minimize selection bias by employing community based study with probability sampling method. Additionally, recall bias was attempted to be reduced by involving women who have given birth in the last 12 months preceding the study.

On the other hand, its limitation is associated with not ascribing the direction of causations to the relationships found in the study because of the nature of cross sectional study design.

## Conclusion

Only a small numbers of respondent were found to be prepared for birth and its complications in their last pregnancy. Place of residence, educational status, ANC follow up, knowledge of key danger signs during pregnancy as well as postpartum period were independent predictors of birth preparedness and complication readiness.

### Recommendation

The study revealed that only a few women were well prepared for birth and its complication. Therefore, ministry of health, Oromia region health bureau, Bale zone health department, Goba woreda health office as well as other partner organizations that are working in maternal health issue should work hard to improve birth preparedness and complication readiness of women.

Educations were found to be one of the predictors of BP/CR. Therefore, Goba woreda health office in collaboration with other stake holders such as Goba woreda education office should further strengthen their effort to empower women with education.

Antenatal care follow up was found to have statistically significant association with birth preparedness and complication readiness. Therefore, health professionals during antenatal care should give due emphasis on birth preparedness and complication readiness plan to improve access to skilled and emergency obstetric care.

Even though majority of women attended ANC, only very small numbers of the respondents were prepared for birth and its complication. Therefore, any interested researcher should conduct further study on quality of ANC in focus of birth preparedness and complication readiness to assess whether health professionals appropriately advise and provide health information concerning birth preparedness and complication readiness.
